# Dipyridamole decreases dialysis risk and improves survival in patients with pre-dialysis advanced chronic kidney disease

**DOI:** 10.18632/oncotarget.19850

**Published:** 2017-08-03

**Authors:** Ko-Lin Kuo, Szu-Chun Hung, Wei-Cheng Tseng, Jia-Sin Liu, Ming-Huang Lin, Chih-Cheng Hsu, Der-Cherng Tarng

**Affiliations:** ^1^ Division of Nephrology, Taipei Tzu Chi Hospital, Buddhist Tzu Chi Medical Foundation, Hualien, Taiwan; ^2^ School of Medicine, Tzu Chi University, Hualien, Taiwan; ^3^ Division of Nephrology, Department of Medicine, Taipei Veterans General Hospital, Taipei, Taiwan; ^4^ Institute of Clinical Medicine and Faculty of Medicine, National Yang-Ming University, Taipei, Taiwan; ^5^ Department of Public Health, Kaohsiung Medical University, Kaohsiung, Taiwan; ^6^ Institute of Population Health Sciences, National Health Research Institutes, Zhunan, Taiwan; ^7^ Department of Health Services Administration, China Medical University, Taichung, Taiwan; ^8^ Department and Institute of Physiology, National Yang-Ming University, Taipei, Taiwan

**Keywords:** chronic kidney disease, dialysis, erythropoiesis-stimulating agent, dipyridamole, renin-angiotensin-aldosterone system blockade

## Abstract

**Introduction:**

Dipyridamole decreases proteinuria and improves renal function progression in patients with glomerular disease through its inhibition of platelet activation and enhanced nitric oxide expression. Few studies have evaluated the effects of dipyridamole on renal outcome or survival in CKD stage 5 patients who have not yet received dialysis (CKD 5 ND).

**Materials and Methods:**

A prospective cohort study was conducted based on the Taiwan National Health Insurance Research Database. From January 1, 2000 to June 30, 2009, we enrolled 28,497 patients who had a serum creatinine > 6 mg/dL and a hematocrit < 28% and who were treated with erythropoiesis-stimulating agents (ESAs). All patients were further divided into two groups with or without dipyridamole use within 90 days after starting ESA therapy. Patient followed-up took place until dialysis, death before initiation of dialysis or December 31, 2009. The primary outcomes were long-term dialysis and death before initiating dialysis.

**Results:**

The dipyridamole users and nonusers groups included 7,746 and 20,751 patients, respectively. We found that 20,152 patients (70.7%) required long-term dialysis and 5,697 patients (20.0%) died before a progression to end-stage renal disease required dialysis. After propensity score-matching, dipyridamole users were associated with lower risks for long-term dialysis (adjusted HR, 0.96; 95% CI, 0.93–0.99) and death (adjusted HR, 0.91; 95% CI, 0.85–0.97) compared with nonusers.

**Conclusions:**

Dipyridamole exhibited a protective effect in reducing the risk for long-term dialysis and death among CKD 5 ND patients. Randomized studies are needed to validate this association.

## INTRODUCTION

Chronic kidney disease (CKD) has profound impacts on public health and the economy [1]. Activation of the renin-angiotensin-aldosterone system (RAAS) and the production of growth factors and inflammatory mediators play pivotal roles in CKD progression [2–5]. Cumulative evidence strongly recommends RAAS blockade, primarily with an angiotensin converting enzyme inhibitor (ACEI) or an angiotensin II receptor blocker (ARB), as first-line antihypertensive agents for the treatment of CKD [6]. Inhibition of the RAAS not only delays the progression of CKD both in non-diabetic and diabetic stage 1–3 CKD patients but also in non-diabetic stage 4 CKD patients in randomized control trials [7–12]. Our previous study demonstrated that the use of RAAS blockade in patients with stage 5 CKD who had not yet received dialysis (CKD 5 ND) was associated with a lower risk for long-term dialysis [13]. Most patients eventually progress to end-stage renal disease (ESRD) even after the intensive use of RAAS blockade. Therefore, it is important to find another strategy to arrest CKD progression.

Dipyridamole stimulates nitric oxide action and inhibits platelet aggregation via the inhibition of phosphodiesterase and has an antioxidant effect [14–16]. A number of clinical studies have shown the reno-protective effects of dipyridamole monotherapy or combination therapy with ACEIs, antiplatelet agents or immunosuppressants in the treatment of early CKD in patients with diabetic kidney disease, IgA nephropathy, and membranoproliferative glomerulonephritis [17–19]. However, studies focusing on dipyridamole monotherapy or its interaction with RAAS blockade in patients with CKD 5 ND that have used hard end points, such as ESRD and mortality, are limited.

Based on Taiwan National Health Insurance (NHI) reimbursement regulations, CKD patients who have a serum creatinine concentration of > 6 mg/dL (approximately stage 5 CKD) and a hematocrit of < 28% could receive erythropoiesis stimulating agent (ESA) to keep a hematocrit concentration not exceeding 36%. This policy provides a unique opportunity to evaluate a study cohort with advanced CKD. To extend the current knowledge about dipyridamole therapy to pre-dialysis advanced CKD, we conducted a nationwide, cohort study to evaluate the association between dipyridamole treatment and the risks of long-term dialysis or death and the interaction between dipyridamole treatment and RAAS blockade.

## RESULTS

### Patient characteristics

**Figure 1:**
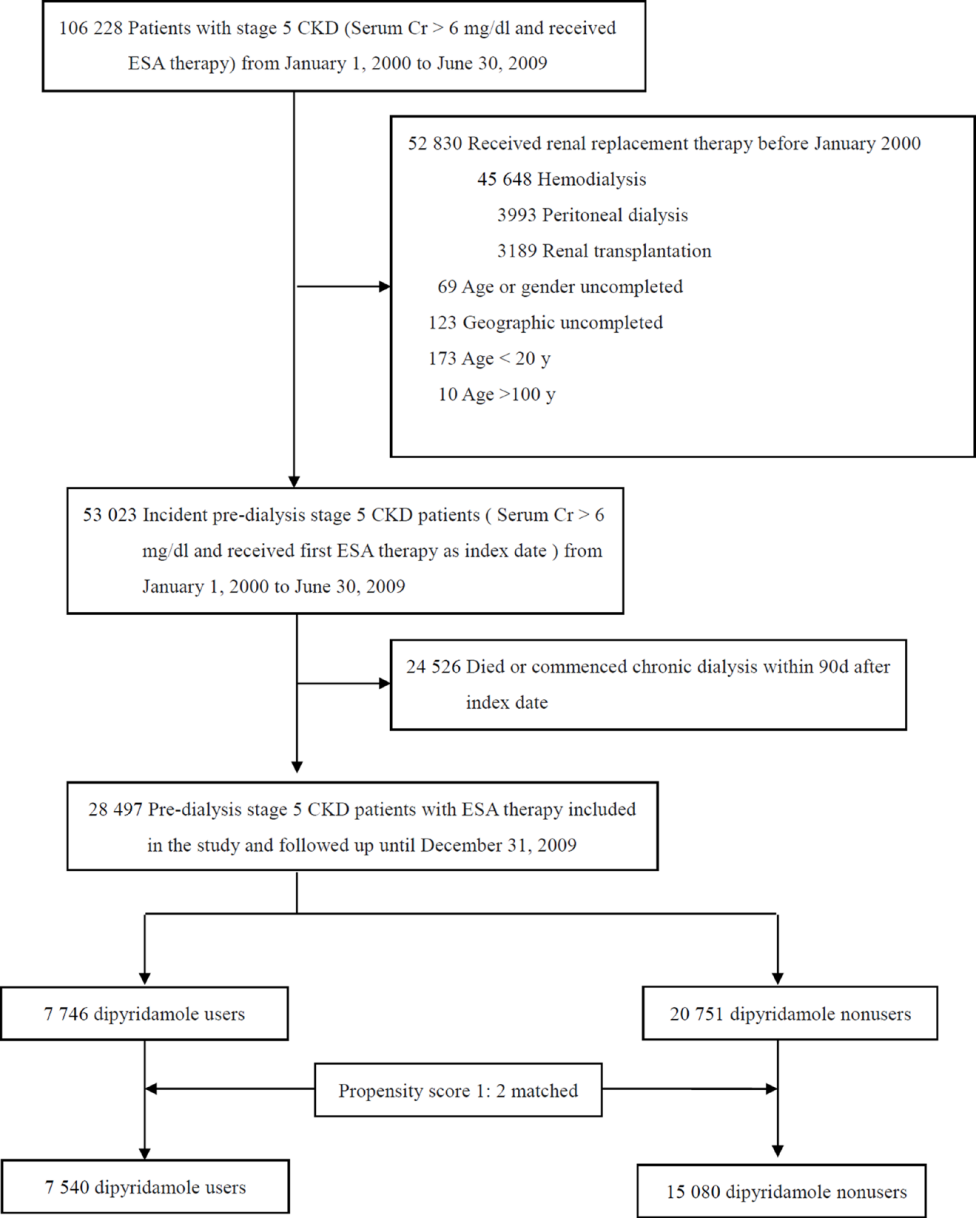
Study profile Abbreviations: CKD, chronic kidney disease; Cr, creatinine; ESA, erythropoiesis stimulating agent.

Figure 1 shows the flow chart for patient selection. The date of the initiation of ESA therapy for each patient was defined as the index date. After excluding those ineligible, we selected 28,497 individuals with CKD 5 ND for further analysis. All patients were classified as dipyridamole users or nonusers within 90 days of the index date. Among this population, 7,746 (27.2%) patients were dipyridamole users and 20,751 (72.8%) were nonusers. The mean age of the dipyridamole users was 66 years, of whom 47.1% were male and 50.6% had diabetes mellitus (Table 1). Compared with the dipyridamole nonusers, the dipyridamole users were older, and less likely to visit nephrologists in the preceding 3 years. Because we expected dipyridamole users and nonusers to differ with respect to prognostic factors confound the outcome analyses, we used a propensity score-based matching to control residual confounding factors. For each dipyridamole user, we identified two nonusers from our selected cohort who has the most similar estimated propensity scores which were calculated from all the baseline characteristics in Table 1. The formula of propensity scores were also listed in Supplementary Table 1. We applied the nearest-neighbor algorithm was to construct matched pairs, assuming that the proportion of 0.995 to 1.0 is perfect [20]. Finally, we matched 7,540 dipyridamole users and 15,080 dipyridamole nonusers (Figure 1).

**Table 1:** Baseline characteristics of study subjects before and after propensity score matching by dipyridamole use

	Before matching				Propensity score-matched			
	Dipyridamole users (*n* =7,746)	Dipyridamole nonusers (*n* = 20,751)	*P*	*SD*	Dipyridamole users (*n* = 7,540)	Dipyridamole nonusers (*n* = 15,080)	*P*	*SD*
Age, mean (SD), y	66 (12.7)	65.2 (13.2)	< 0.001	0.059	65.9 (12.8)	65.9 (13.1)	0.82	0.003
Age group, y								
20–44	431 (5.6)	1,445 (7.0)	< 0.001	0.058	430 (5.7)	975 (6.5)	0.03	0.032
45–64	2,773 (35.8)	7,793 (37.6)	0.006	0.036	2,712 (36.0)	5,361 (35.6)	0.54	0.009
65–74	2,385 (30.8)	5,933 (28.6)	0.001	0.048	2,317 (30.7)	4,427 (29.4)	0.03	0.030
75–100	2,157 (27.9)	5,580 (26.9)	0.11	0.022	2,081 (27.6)	4,317 (28.6)	0.11	0.023
Gender								
Male, *n* (%)	3,645 (47.1)	9,647 (46.5)	0.39	0.012	3,529 (46.8)	7,055 (46.8)	0.98	0.000
Comorbid conditions within the 3 y preceding the index date								
Diabetes, *n* (%)	3,921 (50.6)	11,171 (53.8)	< 0.001	0.064	3,856 (51.1)	7,672 (50.9)	0.71	0.005
MI, *n* (%)	2,134 (27.6)	5,110 (24.6)	< 0.001	0.067	2,027 (26.9)	4,030 (26.7)	0.80	0.004
Stroke, *n* (%)	1,420 (18.3)	3,745 (18.0)	0.57	0.007	1,387 (18.4)	2,780 (18.4)	0.94	0.001
Cancer, *n* (%)	646 (8.3)	2,011 (9.7)	< 0.001	0.047	644 (8.5)	1,244 (8.2)	0.45	0.011
Charlson Comorbidity Index score								
≤ 3, *n* (%)	2,903 (37.5)	7,929 (38.2)	0.26	0.015	2,853 (37.8)	5,754 (38.2)	0.64	0.007
4–5, *n* (%)	1,392 (18.0)	3,814 (18.4)	0.43	0.011	1,363 (18.1)	2,665 (17.7)	0.45	0.011
> 5, *n* (%)	3,451 (44.6)	9,008 (43.4)	0.08	0.023	3,324 (44.1)	6,661 (44.2)	0.90	0.002
Mean (SD)	4.4 (2.3)	4.3 (2.3)	0.001	0.043	4.4 (2.2)	4.4 (2.3)	0.89	0.002
Nephrologist visits within the 3 y preceding the index date								
0, *n* (%)	1,629 (21.0)	4,090 (19.7)	0.013	0.033	1,558 (20.7)	3,129 (20.7)	0.88	0.002
1–6, *n* (%)	2,056 (26.5)	5,562 (26.8)	0.66	0.006	2,007 (26.6)	3,983 (26.4)	0.74	0.005
> 6, *n* (%)	4,061 (52.4)	11,098 (53.5)	0.11	0.021	3,975 (52.7)	7,968 (52.8)	0.87	0.002
Anti-hypertensive agents used								
ACEI, *n* (%)	1,873 (24.2)	4,041 (19.5)	< 0.001	0.114	1,696 (22.5)	3,450 (22.9)	0.52	0.009
ARB, *n* (%)	2,634 (34.0)	7,673 (37.0)	< 0.001	0.062	2,611 (34.6)	5,157 (34.2)	0.52	0.009
Beta-blockers,*n* (%)	3,192 (41.2)	8,898 (42.9)	0.01	0.034	3,123 (41.4)	6,236 (41.4)	0.92	0.001
Calcium channel blockers, *n* (%)	5,915 (76.4)	15,955 (76.9)	0.35	0.012	5,768 (76.5)	11,515 (76.4)	0.82	0.003
Diuretics, *n* (%)	4,817 (62.2)	13,446 (64.8)	< 0.001	0.054	4,741 (62.9)	9,423 (62.5)	0.57	0.008
Pentoxifylline, *n* (%)	1,036 (13.4)	3,398 (16.4)	< 0.001	0.084	1,034 (13.7)	2,009 (13.3)	0.42	0.011
Insulin, *n* (%)	1,679 (21.7)	5,225 (25.2)	< 0.001	0.083	1,671 (22.0)	3,321 (22.0)	0.81	0.003
Statin, *n* (%)	1,275 (16.5)	3,681 (17.7)	0.01	0.034	1,263 (16.8)	2,492 (16.4)	0.67	0.006
Aspirin, *n* (%)	1,556 (20.1)	4,194 (20.2)	0.82	0.003	1,520 (20.2)	3,057 (20.3)	0.84	0.003
Acetaminophen,*n* (%)	3,960 (51.1)	10,704 (51.6)	0.49	0.009	3,861 (51.1)	7,753 (51.3)	0.92	0.001
NSAIDs, *n* (%)								
COX-2 inhibitors	387 (5.0)	932 (4.5)	0.07	0.024	375 (5.0)	765 (5.1)	0.75	0.005
Non-COX-2 inhibitors	2,882 (37.2)	7,198 (34.7)	< 0.001	0.053	2,751 (36.5)	5,543 (36.8)	0.69	0.006
Geographic location								
Northern, *n* (%)	3,251 (42.0)	9,017 (43.5)	0.025	0.030	3,235 (42.9)	6,466 (42.9)	0.97	0.001
Middle, *n* (%)	2,034 (26.3)	4,183 (20.2)	< 0.001	0.145	1,847 (24.5)	3.711 (24.6)	0.85	0.003
Southern, *n* (%)	2,349 (30.3)	7,110 (34.3)	< 0.001	0.084	2,347 (30.9)	4.685 (31.1)	0.93	0.001
Eastern or other islands, *n* (%)	111 (1.4)	442 (2.1)	0.001	0.053	111 (1.5)	218 (1.4)	0.88	0.002
Propensity score	0.733 (0.056)	0.716 (0.058)	< 0.001	0.285	0.721 (0.053)	0.719 (0.055)	0.13	0.021

ACEI = angiotensin converting enzyme inhibitor. ARB =angiotensin II receptor blocker. CCI = Charlson comorbidity index. CKD = chronic kidney disease. COX-2 = cyclooxygenase -2. MI = myocardial infarction. NSAID = non-steroidal anti-inflammatory drug. SD = standardized difference.

### Protective effects of dipyridamole in patients with advanced CKD

**Figure 2:**
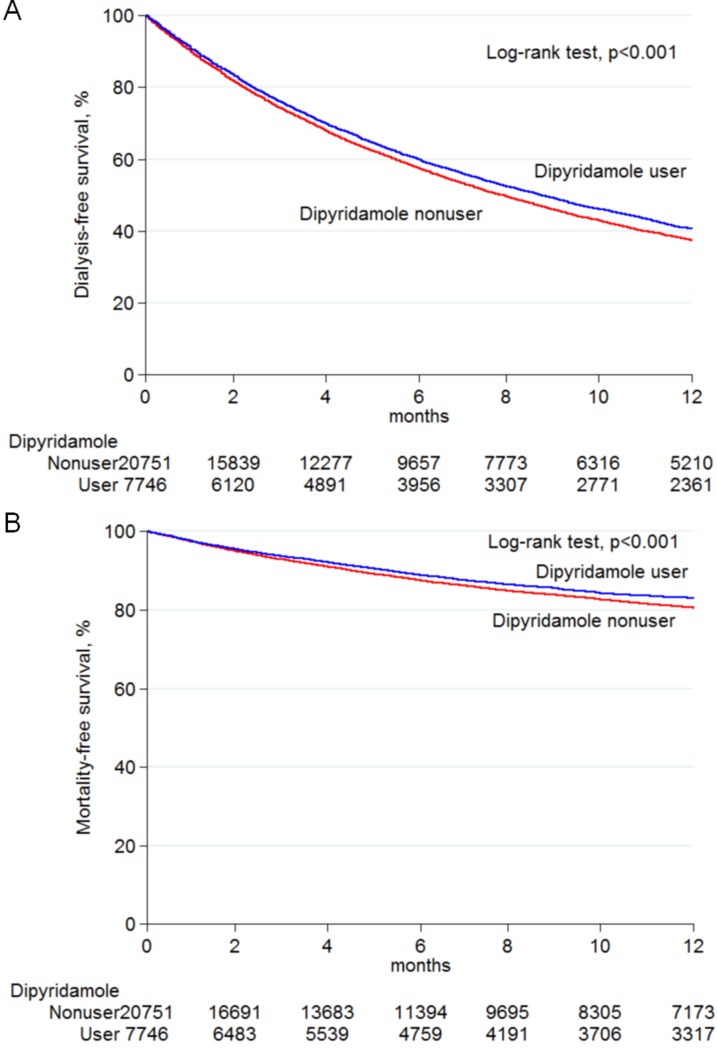
Kaplan–Meier analysis of survival curves among pre-dialysis stage 5 CKD patients Dialysis-free (**A**) and mortality-free (**B**) survivals constitute the study end points. Difference between dipyridamole users and non-users was analyzed by log-rank test. Abbreviations: CKD, chronic kidney disease.

During the study period, the total follow-up summation was 30,143 person-years. The mean follow-up time was 13.9 months in the dipyridamole users and 12.5 months in the nonusers. A total of 20,152 (70.7%) patients progressed to ESRD, necessitating long-term dialysis, and 5,697 (20.0%) died before a progression to ESRD required long-term dialysis (Table 2). The incidence of long-term dialysis was 69.7 per 100 person-years in the dipyridamole users and 72.5 per 100 person-years in the nonusers. The Kaplan-Meier survival curve revealed that patients treated with dipyridamole exhibited a significantly decreased risk of requiring chronic dialysis (Figure 2A). Compared with the nonusers, the dipyridamole users exhibited a lower chance of progression to ESRD requiring maintenance dialysis (adjusted HR, 0.97; 95% CI 0.94–1.00), and the results remained consistent after propensity score-matching (adjusted HR, 0.96; 95% CI, 0.93–0.99) (Table 2). On the dose-response relationship, we found that respective HRs of long-term dialysis related to dipyridamole use were significantly lower in cumulative defined daily doses (DDDs) ≥ 140 within 90 days (adjusted HR, 0.91; 95% CI, 0.87–0.95) or a prescribed daily dose of ≥ 75 mg (adjusted HR, 0.91; 95% CI, 0.88–0.95) compared to the dipyridamole nonusers (Table 2).

**Table 2:** Risk of long-term dialysis and death among patients with advanced CKD comparing dipyridamole users vs. nonusers

	Before Matching				After Matching		
	*N* of event	Incidence rate (100 person- years)	Crude HR (95% CI)	Adjusted HR (95% CI)	*N* of event	Incidence rate (100 person- years)	Adjusted HR (95% CI)
**Long-term dialysis**							
Dipyridamole nonuser	14,463	74.26	1.0 (reference)	1.0 (reference)	10,480	72.48	1.0 (reference)
Dipyridamole user	5,689	69.35	0.94 (0.91–0.97)^*^	0.97 (0.94–1.00)^*^	5,531	69.73	0.96 (0.93–0.99)^*^
< 140 DDD	2,768	72.49	0.98 (0.94–1.02)	1.02 (0.98–1.06)	2,695	73.18	1.02 (0.98–1.06)
≥ 140 DDD	2,921	66.61	0.91 (0.87–0.95)^*^	0.92 (0.88–0.96)^*^	2,836	66.74	0.91 (0.87–0.95)^*^
< 75 mg/day	2,617	72.90	0.99 (0.95–1.03)	1.02 (0.98–1.07)	2,546	73.66	1.03 (0.98–1.07)
≥ 75 mg/day	3,072	66.58	0.91 (0.87–0.94)^*^	0.92 (0.89–0.96)^*^	2,985	66.70	0.91 (0.88–0.95)^*^
**Death**							
Dipyridamole nonuser	4,116	21.13	1.0 (reference)	1.0 (reference)	3,087	21.35	1.0 (reference)
Dipyridamole user	1,581	19.27	0.91 (0.86–0.96)^*^	0.90 (0.85–0.95)^*^	1,536	19.37	0.91 (0.85–0.97)^*^
< 140 DDD	828	21.69	1.02 (0.95–1.10)	0.95 (0.88–1.02)	800	21.72	0.95 (0.88–1.03)
≥ 140 DDD	753	17.17	0.81 (0.75–0.87)^*^	0.85 (0.79–0.92)^*^	736	17.32	0.86 (0.80–0.94)^*^
< 75 mg/day	781	21.76	1.03 (0.95–1.11)	0.94 (0.87–1.02)	754	21.81	0.95 (0.87–1.03)
≥ 75 mg/day	800	17.34	0.82 (0.76–0.88)^*^	0.86 (0.80–0.93)^*^	782	17.47	0.87 (0.81–0.95)^*^

CI = confidence interval. CKD = chronic kidney disease. DDD = defined daily doses. HR = hazard ratio

A multivariate analysis was adjusted for all variables listed in Table 1.

^*^P < 0.05 compared with dipyridamole nonusers

**Figure 3:**
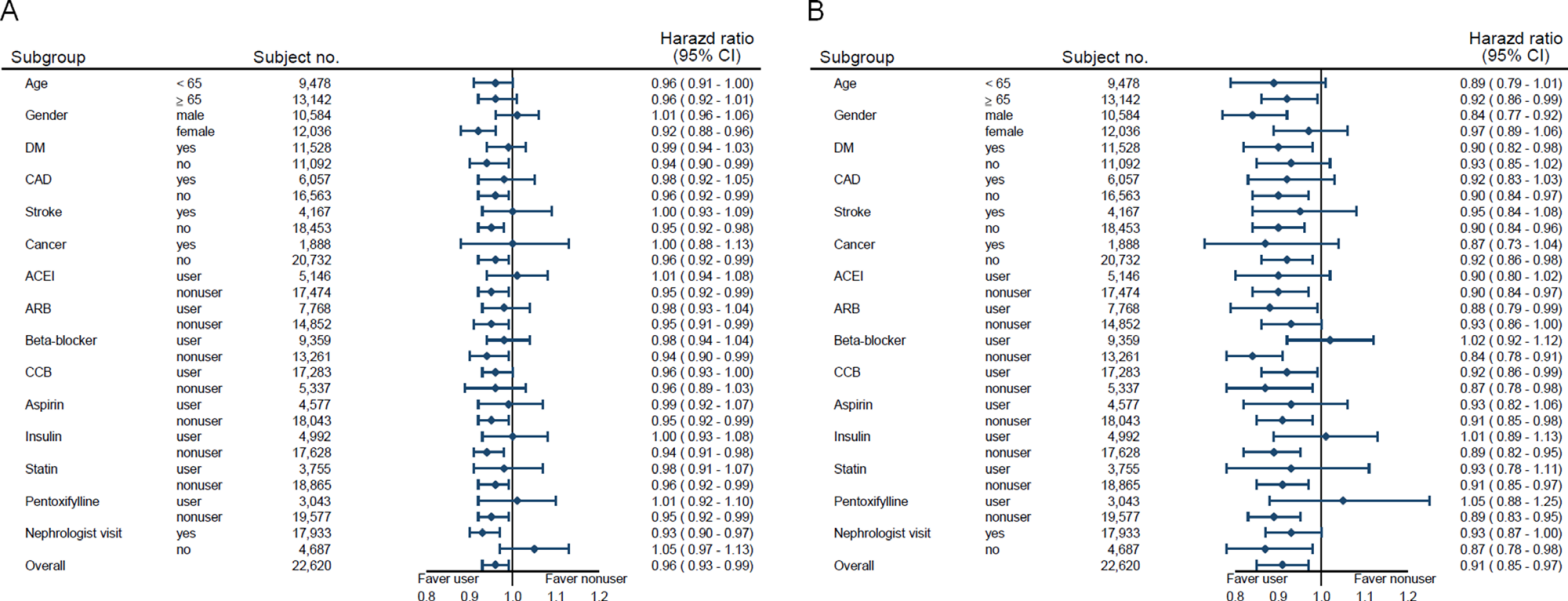
Adjusted hazard ratios of long-term dialysis (**A**) and death (**B**) among pre-dialysis stage 5 CKD patients by dipyridamole use. Abbreviations; ACEI, angiotensin converting enzyme inhibitor; ARB, angiotensin II receptor blocker; CAD, coronary artery disease; CCB, calcium channel blocker; CI, confidence interval; CKD, chronic kidney disease; DM, diabetes mellitus; HR, hazard ratios.

Table 2 shows the association between dipyridamole administration and pre-dialysis death. The incidence of death was 19.4 per 100 person-years in the dipyridamole users and 21.4 per 100 person-years in the nonusers. The Kaplan-Meier survival curve disclosed that the dipyridamole users had a significantly lower chance of death (Figure 2B). Compared with the nonusers, the dipyridamole users exhibited a decreased risk of death (adjusted HR, 0.90; 95% CI 0.85–0.95), and the results remained consistent after propensity score-matching (adjusted HR, 0.91; 95% CI, 0.85–0.97) (Table 2). On the dose-response relationship, we found that the respective HRs of pre-dialysis death related to dipyridamole use were significantly lower in cumulative DDDs ≥ 140 within 90 days (adjusted HR, 0.86; 95% CI, 0.80–0.94) or a prescribed daily dose of ≥ 75 mg (adjusted HR, 0.87; 95% CI, 0.81–0.95) compared to the dipyridamole nonusers (Table 2). In the stratified analyses, the decreased HRs of chronic dialysis and death in the dipyridamole cohort were generally consistent across nearly all subgroups (Figure 3). Finally, in the sensitivity analyses, the results were consistent with the main findings. The estimated effects of dipyridamole use on the primary outcomes were similar regardless of different exposure time for dipyridamole use calculated (Supplementary Tables 2–4).

### Interaction of dipyridamole and RAAS blockade on the risk of long-term dialysis and death in CKD 5 ND patients

**Figure 4:**
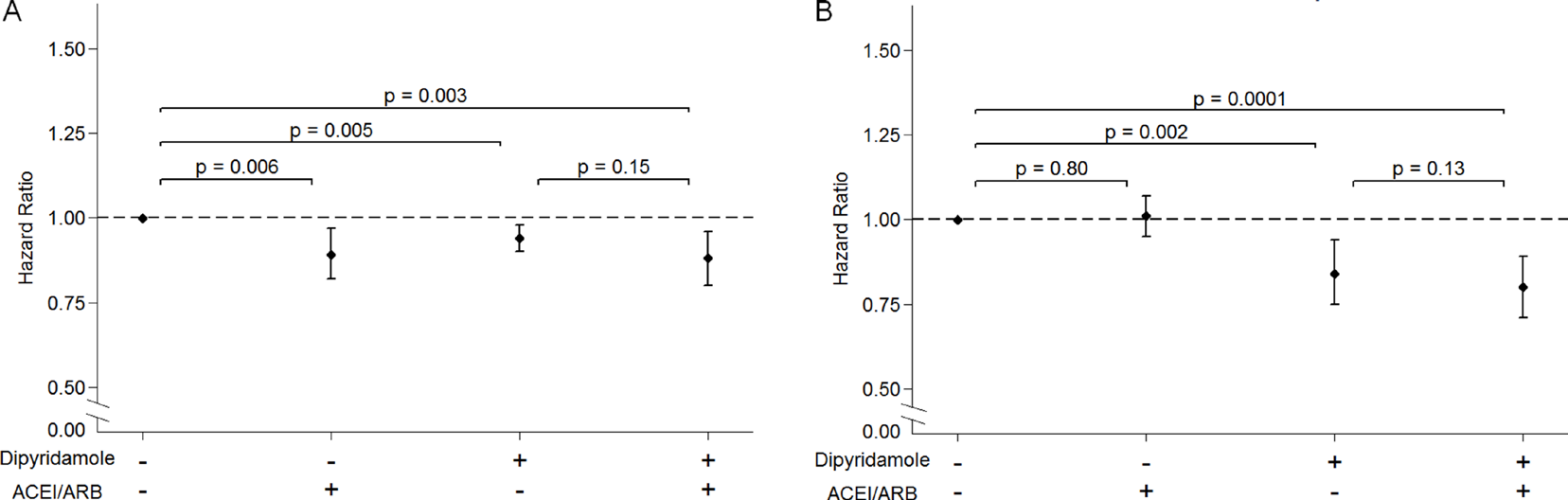
The interaction of dipyridamole and the renin-angiotensin-aldosterone system blockade on the risk of long-term dialysis (**A**) and death (**B**) among pre-dialysis stage 5 CKD patients. Abbreviations; ACEI, angiotensin converting enzyme inhibitor; ARB, angiotensin II receptor blocker; HR, hazard ratios.

In the assessment of the interaction between dipyridamole and ACEI/ARB, we found that either ACEI/ARB or dipyridamole administration was significantly associated with a lower risk for chronic dialysis compared to the patients who had taken neither ACEI/ARB nor dipyridamole. Moreover, concurrent use of an ACEI/ARB and dipyridamole significantly diminished the risk of progression to ESRD (Figure 4A). In addition, either dipyridamole monotherapy or the concurrent use of ACEI/ARB was significantly associated with a lower risk of death compared to those not taking ACEI/ARB and dipyridamole. However, ACEI/ARB use alone was not significantly associated with a lower risk of death (Figure 4B).

### Risk of bleeding in CKD 5 ND patients receiving dipyridamole

During the median follow-up period of 17.8 months, a total of 3,615 (12.7%) patients had bleeding events (Supplementary Table 5). Compared with the dipyridamole nonusers, the risk of bleeding in dipyridamole users did not significantly increase (adjusted HR, 0.99; 95% CI 0.92–1.06). After the propensity score-matching, the dipyridamole users still did not have a significantly higher chance for bleeding (adjusted HR, 1.00; 95% CI, 0.92–1.08).

## DISCUSSION

The mechanism of the reno-protective effects of dipyridamole has been explained by several animal and human studies. Dipyridamole increased the local concentration of adenosine, which stimulated adenylyl cyclase in platelets, leading to increased intracellular levels of cyclic adenosine monophosphate (cAMP) [21]. By inhibiting phosphodiesterase (PDE), dipyridamole increases prostacyclin (PGI_2_) production and cyclic guanine monophosphate (cGMP) levels on vascular smooth muscle, leading to vasodilation [22, 23]. Moreover, by increasing intracellular levels of cGMP, dipyridamole can augment many of the downstream signaling pathways of nitric oxide [14]. Hewitson *et al.* disclosed that dipyridamole inhibits profibrotic activities of renal fibroblasts and collagen synthesis *in vitro* [24]. In animal models, dipyridamole monotherapy or combinations with ACEI therapy attenuated microalbuminuria and enhanced eNOS expression in streptozotocin-induced diabetic rats [25]. In rats with subtotal nephrectomy, dipyridamole or ACEI therapy markedly improved renal function. Further histological examination of the remnant kidney detected the presence of vasodilation with a lower degree of podocyte swelling in both dipyridamole and ACEI treatment groups. These data indicated that dipyridamole still attenuated the progression of glomerular disease in advanced CKD in rats [26]. In humans, dipyridamole monotherapy reduced urinary albumin excretion in diabetes patients with normo- or microalbuminuria [17]. In two meta-analyses of antiplatelet therapy for IgA nephropathy, dipyridamole therapy was beneficial for reducing the risk of proteinuria [27, 28]. In addition, dipyridamole combination therapy with ACE-I, antiplatelet agents or immunosuppressants significantly reduced proteinuria in patients with IgA nephropathy and primary membranoproliferative glomerulonephritis [18, 19, 29, 30]. However, our knowledge about the reno-protective effect of dipyridamole use is limited in pre-dialysis, advanced CKD patients.

Limited studies have disclosed the long-term outcome of dipyridamole use other than its effects on decreasing proteinuria or hastening the GFR decline rate in CKD patients who had not yet received dialysis. This is not surprising, because the previous studies were small in scale, and all the study periods were short. To our knowledge, our national cohort study first demonstrated that dipyridamole treatment was associated with a 4% lower risk of long-term dialysis and a 9% lower risk of death in CKD 5 ND patients. Moreover, the risk of bleeding events was not significantly increased in dipyridamole users. A small-scale observational study demonstrated that the use of dipyridamole provided a better renal outcome in CKD patients with a mean eGFR of 25.5 ml/minute/1.73 m^2^ [31]. The authors also showed that dipyridamole users exhibited a decreased risk of death compared to non-users. Whether their result could be extrapolated to stage 5 ND was questionable. In contrast, our study not only extends the current knowledge in the field but also demonstrates the consistency and generalizability of the effectiveness of dipyridamole in patients with early stage CKD to CKD 5 ND.

From a clinical viewpoint, several issues merit discussion in this study. First, for patients with rapidly declining renal function and low GFR, such as CKD 5 ND patients, physicians usually do not prescribe RAAS blockade, and these patients were excluded in many studies. This is the reason why research about medications in pre-dialysis advanced CKD patients is limited. However, our previous study demonstrated for the first time that ACEI/ARB users exhibited a 6% lower risk of long-term dialysis or death. In the present study, we demonstrated that dipyridamole represents a promising and safe agent for reno-protection, either as monotherapy or in combination with RAAS blockade. Second, some CKD patients cannot tolerate ACEI/ARB use because of hypotension, hyperkalemia or renal artery stenosis. Dipyridamole treatment might be a better choice for reno-protection in these situations. Third, we found that dipyridamole monotherapy, but not RAAS blockade, was associated with low death risk in CKD 5 ND patients. The possible causes might be related to the stimulation of nitric oxide action and the inhibition of platelet aggregation via the inhibition of phosphodiesterase and the antioxidant effect of dipyridamole [14–16]. Our results indicated that these platelet and non-platelet actions of dipyridamole may contribute to its translational therapeutic benefits not only in patients with traditional vascular disease but also in patients with advanced CKD.

Some limitations should be addressed. First, since our cohort study is observational, it cannot prove causality. However, to conduct a randomized controlled trial with adequate statistical power (*α* = 0.05; 1-*β* = 0.8), we needed recruiting at least 21,806 CKD 5 ND patients to examine a 9% relative risk reduction of death. It seems impracticable to conduct such a large-scale trial in this population. Moreover, some researches reveal that well-designed observational cohort studies may generate comparable outcomes [32, 33]. Second, although some risk factors for predicting CKD progression, such as proteinuria or renal function, are not available in the present study. We did a propensity score-based matched design to minimize these confounders. In addition, the findings of sensitivity and subgroup analyses generally support the robustness of our results; however, neutral effects of dipyridamole use could be seen in some subgroup analyses, indicating we rather be more conservative to make our conclusion. Randomized clinical trials are needed to validate our findings in the future. Moreover, nephrologists usually prescribe dipyridamole in patients with proteinuria, the use of dipyridamole in patients with more proteinuria will bias the result toward the null hypothesis. Third, some patients with transient creatinine concentrations of > 6 mg/dL might be recruited in this cohort. Therefore, we had restricted our analysis to subjects who received ESA at least two consecutive visits, and the result remained unchanged. Finally, the generalizability of our study is limited to advanced CKD patients who are anemic. Our results can't be applicable to all stage 5 CKD patients who had not yet received dialysis.

In conclusion, our nationwide cohort study extend the knowledge of dipyridamole therapy from early-stage CKD patients to CKD 5 ND patients. Our study reveals that either dipyridamole monotherapy or the combination use of dipyridamole and RAAS blockade were significantly associated with a decreased risk of long-term dialysis and pre-dialysis death in CKD 5 ND patients. Moreover, the risk of bleeding events was not significantly increased in dipyridamole users. Further randomized controlled studies may be needed to provide definitive results.

## MATERIALS AND METHODS

### Data source

Patient's data were retrieved from Taiwan NHI Research Database, which contains the health-care data gathered prospectively for 99% of the entire population of 23 million people [34]. De-identified information recorded in the NHI Research Database includes diagnostic codes according to the International Classification of Diseases-9th revision (ICD-9), birthday, gender, residency area, drug prescriptions, and medical procedures. The study methods were carried out in accordance with the approved guidelines for research involving human subjects from the Taiwan Ministry of Health and Welfare. The Institutional Review Board at Taipei Tzu Chi Hospital approved the study. Informed consent was waived due to the de-identification of any personal information in this database.

### Design and study participants

We selected patients who had CKD and received ESA therapy in the NHI Research Database between January 1, 2000 and June 30, 2009, and then were followed up until December 31, 2009. Taiwan NHI reimbursement regulations state that ESA treatment can be initiated when patients with CKD who do not need dialysis have a serum creatinine concentration of > 6 mg/dL (approximately equivalent to an estimated GFR of < 15 ml/min per 1.73 m^2^) and a hematocrit of < 28%, to maintain a hematocrit not exceeding 36%. The selected cohort had been described in our previous study [13, 35]. According to the record by the Taiwan Ministry of Health and Welfare, 85% of CKD 5 ND patients received a prescription for ESA. Therefore, this indicated that our selected cohort was highly representative of patients with stage 5 CKD in Taiwan [36]. We defined the first day of ESA administration as the index date. Our study excluded individuals younger than 20 years or older than 100 years, those who received dialysis or kidney transplantation before treatment with ESA, and individuals who died or who begun renal replacement therapy within 90 days of first dose of ESA prescription. We defined comorbid disorders, including diabetes, myocardial infarction, stroke, and cancer, which had been diagnosed within 3 years preceding the index date. We used Charlson comorbidity index (CCI) to quantify patients’ comorbidity profiles [37].

### Exposure assessment

Patients who had taken dipyridamole within 90 days of the index date were defined as dipyridamole users (*n* = 7,746), and the remaining subjects were defined as dipyridamole nonusers (*n* = 20,751). Furthermore, to assess dose-effect, we analyzed the risk of chronic dialysis and death according to the cumulative DDD during the 90-day exposure period (< 140 DDD, ≥ 140 DDD) and the prescribed daily dose (< 75 mg, ≥ 75 mg), relative to no dipyridamole use. DDD, a technical unit of measurement, is defined as the assumed average maintenance dose per day for a drug used for its main indication in adults, as previously described [35].

### Renal outcome and mortality

The observation period begun 90 days after the index date until the initiation of maintenance dialysis, death, or December 31, 2009, whichever happened first. The primary outcomes were long-term dialysis or pre-dialysis death. The onset of renal outcome was defined as the date of initiation of long-term dialysis for at least 90 days. The onset of mortality was defined as the date of death.

### Statistical analysis

The baseline characteristics were compared with the 2-sided *t* test and the *chi-square* test. We defined the study entry as the 90th day after the index date. Patient follow-up visits happened until the time of long-term dialysis, death or December 31, 2009. The primary outcomes were long-term chronic dialysis and pre-dialysis death. We used Cox's proportional hazard models to compare renal outcome and death while controlling for baseline covariates. We expressed results as HRs with 95% CIs, compared with dipyridamole nonusers. We evaluated proportional hazard assumption by comparing estimated log-log survival curves for all time-independent covariates. Because we expected dipyridamole users and nonusers to differ with respect to prognostic factors confound the outcome analyses, we used a propensity score-based matching to control residual confounding factors. We incorporated all baseline characteristics listed in Table 1 into our analysis as independent variables. We deemed a two-sided *P* values less than 0.05 significant. We performed statistical analyses with SAS version 9.3, and STATA SE version 14.

### Subgroup and sensitivity analyses

To evaluate effect modification, we did subgroup analyses in pre-specified strata of clinical interest, including age, gender, the presence or absence of diabetes mellitus, CAD, stroke, cancer, the use of anti-hypertensive medications, aspirin, insulin, statin and pentoxifylline use, and nephrologist care. To assess the reliability of our findings, we performed a series of analyses to define dipyridamole administration at intervals of 30, 60 days and 120 days after first ESA use to minimize misclassification bias (Supplementary Tables 2–4).

## SUPPLEMENTARY MATERIALS TABLES


