# Supplementary material for: Dipyridamole decreases dialysis risk and improves survival in patients with pre-dialysis advanced chronic kidney disease
Source: Oncotarget. 2017 Aug 3;9(4):5368–77. doi: 10.18632/oncotarget.19850 (PMC5797055; doi:10.18632/oncotarget.19850)
Supplement: Supplementary file 1 [file oncotarget-09-5368-s001.pdf]

# Dipyridamole decreases dialysis risk and improves survival in patients with pre-dialysis advanced chronic kidney disease

## SUPPLEMENTARY MATERIALS

### Supplementary Table 1: The formula of propensity scores in logistic model

| | coefficient | Standard error | Odds ratio | 95% lower confidence interval | 95% upper confidence interval | *P* value |
|---|---|---|---|---|---|---|
| Age, y | -0.00305 | 0.0011 | 0.997 | 0.995 | 0.999 | 0.0056 |
| Male vs. female | -0.039 | 0.0279 | 0.962 | 0.911 | 1.016 | 0.1621 |
| Comorbid condition with in the 3 preceding the index date | | | | | | |
| Diabetes | 0.112 | 0.0336 | 1.119 | 1.047 | 1.195 | 0.0009 |
| MI | -0.1343 | 0.0325 | 0.874 | 0.82 | 0.932 | <.0001 |
| Stroke | 0.0421 | 0.0376 | 1.043 | 0.969 | 1.123 | 0.2629 |
| Cancer | 0.2743 | 0.0503 | 1.316 | 1.192 | 1.452 | <.0001 |
| CCI score | -0.0435 | 0.0074 | 0.957 | 0.944 | 0.971 | <.0001 |
| Nephrologist visits within the 3 y preceding the index date | | | | | | |
| 0 | 1 | 1 | 1 | - | - | |
| 1–6 | 0.0635 | 0.0399 | 1.066 | 0.985 | 1.152 | 0.1115 |
| > 6 | 0.0734 | 0.0361 | 1.076 | 1.003 | 1.155 | 0.0418 |
| Anti-hypertensive agents used | | | | | | |
| ACEI | -0.2652 | 0.0326 | 0.767 | 0.72 | 0.818 | <.0001 |
| ARB | 0.093 | 0.0287 | 1.097 | 1.037 | 1.161 | 0.0012 |
| Beta-blockers | -0.0219 | 0.0326 | 0.978 | 0.918 | 1.043 | 0.5004 |
| Calcium channel blockers | 0.0553 | 0.0283 | 1.057 | 1 | 1.117 | 0.0504 |
| Diuretics | -0.0239 | 0.0326 | 0.976 | 0.916 | 1.041 | 0.464 |
| Pentoxifylline | 0.2839 | 0.0394 | 1.328 | 1.23 | 1.435 | <.0001 |
| Insulin | 0.2212 | 0.0368 | 1.248 | 1.161 | 1.341 | <.0001 |
| Statin | -0.0232 | 0.0378 | 0.977 | 0.907 | 1.052 | 0.5402 |
| Aspirin | 0.0184 | 0.0358 | 1.019 | 0.949 | 1.093 | 0.6077 |
| Acetaminophen | 0.0147 | 0.0284 | 1.015 | 0.96 | 1.073 | 0.6043 |
| NSAIDs | | | | | | |
| COX-2 inhibitors | -0.1039 | 0.0298 | 0.901 | 0.85 | 0.956 | 0.0005 |
| Non-COX-2 inhibitors | -0.0428 | 0.0636 | 0.958 | 0.846 | 1.085 | 0.501 |
| Geographic location | | | | | | |
| Northern | 1 | 1 | 1 | - | - | |
| Middle | -0.274 | 0.0346 | 0.76 | 0.71 | 0.814 | <.0001 |
| Southern | 0.1503 | 0.0323 | 1.162 | 1.091 | 1.238 | <.0001 |
| Eastern or other islands | 0.405 | 0.109 | 1.499 | 1.211 | 1.856 | 0.0002 |

ACEI = angiotensin converting enzyme inhibitor. ARB =angiotensin II receptor blocker. CCI = Charlson comorbidity index. COX-2 = cyclooxygenase -2. MI = myocardial infarction. NSAID = non-steroidal anti-inflammatory drug.

## Supplementary Table 2: Risks of long-term dialysis and death in pre-dialysis stage 5 CKD patients with dipyridamole exposure within 30 days and observation period beginning 30 days after the index date

| | Before Matching | | | | After Matching | | |
|---|---|---|---|---|---|---|---|
| | *N* of events | Incidence rate (100 person- years) | Crude HR (95% CI) | Adjusted HR (95% CI) | *N* of events | Incidence rate (100 person- years) | Adjusted HR (95% CI) |
| **Long-term dialysis** | | | | | | | |
| Dipyridamole nonuser | 19,622 | 79.94 | 1.0 (reference) | 1.0 (reference) | 14,160 | 77.62 | 1.0 (reference) |
| Dipyridamole user | 7,685 | 76.12 | 0.97 (0.94–0.99)* | 0.99 (0.96–1.01) | 7,511 | 76.60 | 0.98 (0.96–1.01) |
| < 140 DDD | 4,086 | 83.83 | 1.06 (1.02–1.10)* | 1.10 (1.06–1.14)* | 3,995 | 84.53 | 1.10 (1.06–1.14)* |
| ≥ 140 DDD | 3,599 | 68.93 | 0.88 (0.85–0.91)* | 0.88 (0.85–0.92)* | 3,516 | 69.22 | 0.88 (0.85–0.91)* |
| < 75 mg/day | 3,908 | 84.98 | 1.08 (1.04–1.11)* | 1.11 (1.07–1.15)* | 3,819 | 85.75 | 1.11 (1.07–1.15)* |
| ≥ 75 mg/day | 3,777 | 68.71 | 0.87 (0.84–0.90)* | 0.88 (0.85–0.92)* | 3,692 | 68.99 | 0.88 (0.85–0.91)* |
| **Death** | | | | | | | |
| Dipyridamole nonuser | 5,327 | 21.70 | 1.0 (reference) | 1.0 (reference) | 4,039 | 22.14 | 1.0 (reference) |
| Dipyridamole user | 1,992 | 19.73 | 0.91 (0.86–0.96)* | 0.89 (0.84–0.93)* | 1,941 | 19.80 | 0.89 (0.84–0.94)* |
| < 140 DDD | 1,160 | 23.80 | 1.10 (1.04–1.18)* | 1.04 (0.97–1.10) | 1,128 | 23.87 | 1.04 (0.97–1.11) |
| ≥ 140 DDD | 832 | 15.93 | 0.73 (0.68–0.78)* | 0.74 (0.69–0.79)* | 813 | 16.01 | 0.74 (0.69–0.80)* |
| < 75 mg/day | 1,101 | 23.94 | 1.11 (1.04–1.19)* | 1.04 (0.97–1.11) | 1,071 | 24.05 | 1.04 (0.97–1.11) |
| ≥ 75 mg/day | 891 | 16.21 | 0.74 (0.69–0.79)* | 0.75 (0.70–0.81)* | 870 | 16.26 | 0.76 (0.70–0.81)* |

Abbreviations: CI, confidence interval; CKD, chronic kidney disease; DDD, defined daily doses; HR, hazard ratio
A multivariate analysis was adjusted for all of the variables listed in Table 1.
*$P < 0.05$ compared with dipyridamole nonusers.

## Supplementary Table 3: Risks of long term dialysis and death in pre-dialysis stage 5 CKD patients with dipyridamole exposure within 60 days and observation period beginning 60 days after the index date

| | Before Matching | | | | After Matching | | |
|---|---|---|---|---|---|---|---|
| | *N* of events | Incidence rate (100 person- years) | Crude HR (95% CI) | Adjusted HR (95% CI) | *N* of events | Incidence rate (100 person- years) | Adjusted HR (95% CI) |
| **Long-term dialysis** | | | | | | | |
| Dipyridamole nonuser | 16,804 | 77.01 | 1.0 (reference) | 1.0 (reference) | 12,199 | 75.69 | 1.0 (reference) |
| Dipyridamole user | 6,614 | 72.82 | 0.96 (0.94–0.99)* | 0.98 (0.95–1.01) | 6,459 | 73.26 | 0.97 (0.94–1.00)* |
| < 140 DDD | 3,329 | 77.61 | 1.01 (0.98–1.05) | 1.06 (1.02–1.10)* | 3,254 | 78.46 | 1.05 (1.01–1.09) |
| ≥ 140 DDD | 3,285 | 68.53 | 0.91 (0.87–0.94)* | 0.91 (0.88–0.95)* | 3,205 | 68.63 | 0.89 (0.86–0.93)* |
| < 75 mg/day | 3,169 | 78.46 | 1.03 (0.99–1.07) | 1.07 (1.03–1.11)* | 3,096 | 79.39 | 1.06 (1.02–1.11) |
| ≥ 75 mg/day | 3,445 | 68.30 | 0.90 (0.87–0.93)* | 0.91 (0.88–0.95)* | 3,363 | 68.39 | 0.89 (0.86–0.93)* |
| **Death** | | | | | | | |
| Dipyridamole nonuser | 4,679 | 21.44 | 1.0 (reference) | 1.0 (reference) | 3,571 | 22.16 | 1.0 (reference) |
| Dipyridamole user | 1,787 | 19.68 | 0.92 (0.87–0.97)* | 0.90 (0.85–0.95)* | 1,739 | 19.72 | 0.89 (0.84–0.94)* |
| < 140 DDD | 979 | 22.83 | 1.07 (1.00–1.14) | 0.99 (0.93–1.07) | 948 | 22.86 | 0.98 (0.91–1.05) |
| ≥ 140 DDD | 808 | 16.86 | 0.78 (0.73–0.84)* | 0.80 (0.75–0.87)* | 791 | 16.94 | 0.80 (0.74–0.86)* |
| < 75 mg/day | 926 | 22.93 | 1.07 (1.00–1.15)* | 0.99 (0.92–1.07) | 897 | 23.00 | 0.98 (0.91–1.04) |
| ≥ 75 mg/day | 861 | 17.07 | 0.79 (0.74–0.85)* | 0.82 (0.76–0.88)* | 842 | 17.12 | 0.81 (0.75–0.87)* |

Abbreviations: CI, confidence interval; CKD, chronic kidney disease; DDD, defined daily doses; HR, hazard ratio
A multivariate analysis was adjusted for all of the variables listed in Table 1.
*$P < 0.05$ compared with dipyridamole nonusers.

## Supplementary Table 4: Risks of long term dialysis and death in pre-dialysis stage 5 CKD patients with dipyridamole exposure within 120 days and observation period beginning 120 days after the index date

| | Before Matching | | | | After Matching | | |
|---|---|---|---|---|---|---|---|
| | *N* of events | Incidence rate (100 person- years) | Crude HR (95% CI) | Adjusted HR (95% CI) | *N* of events | Incidence rate (100 person- years) | Adjusted HR (95% CI) |
| **Long-term dialysis** | | | | | | | |
| Dipyridamole nonuser | 12,423 | 71.29 | 1.0 (reference) | 1.0 (reference) | 9,012 | 68.83 | 1.0 (reference) |
| Dipyridamole user | 4,990 | 67.01 | 0.95 (0.92–0.98)* | 0.97 (0.94–1.00) | 4,836 | 67.66 | 0.98 (0.95–1.01) |
| < 140 DDD | 2,370 | 69.12 | 0.97 (0.93–1.01) | 1.01 (0.97–1.06) | 2,296 | 70.15 | 1.03 (0.98–1.08) |
| ≥ 140 DDD | 2,620 | 65.21 | 0.93 (0.89–0.97)* | 0.93 (0.89–0.97)* | 2,540 | 65.56 | 0.94 (0.90–0.98)* |
| < 75 mg/day | 2,236 | 69.41 | 0.98 (0.93–1.02) | 1.02 (0.97–1.06) | 2,163 | 70.52 | 1.04 (0.99–1.09) |
| ≥ 75 mg/day | 2,754 | 65.18 | 0.92 (0.89–0.96)* | 0.93 (0.89–0.97)* | 2,673 | 65.52 | 0.94 (0.90–0.98)* |
| **Death** | | | | | | | |
| Dipyridamole nonuser | 3,579 | 20.54 | 1.0 (reference) | 1.0 (reference) | 2,768 | 21.14 | 1.0 (reference) |
| Dipyridamole user | 1,389 | 18.65 | 0.90 (0.85–0.96)* | 0.89 (0.84–0.95)* | 1,343 | 18.79 | 0.88 (0.82–0.94)* |
| < 140 DDD | 710 | 20.71 | 1.00 (0.93–1.09) | 0.94 (0.86–1.02) | 681 | 20.81 | 0.92 (0.84–1.00)* |
| ≥ 140 DDD | 679 | 16.90 | 0.81 (0.75–0.88)* | 0.85 (0.78–0.93)* | 662 | 17.09 | 0.85 (0.78–0.92)* |
| < 75 mg/day | 666 | 20.67 | 1.00 (0.92–1.09) | 0.93 (0.85–1.01) | 638 | 20.80 | 0.91 (0.83–0.99)* |
| ≥ 75 mg/day | 723 | 17.11 | 0.83 (0.76–0.89)* | 0.86 (0.80–0.94)* | 705 | 17.28 | 0.86 (0.79–0.93)* |

Abbreviations: CI, confidence interval; CKD, chronic kidney disease; DDD, defined daily doses; HR, hazard ratio
A multivariate analysis was adjusted for all of the variables listed in Table 1.
*$P < 0.05$ compared with dipyridamole nonusers.

## Supplementary Table 5: Risk of bleeding in patients with pre-dialysis stage 5 CKD receiving dipyridamole as compared with dipyridamole nonusers

| | Before Matching | | | | After Matching | | |
|---|---|---|---|---|---|---|---|
| | *N* of event | Incidence rate (100 person- years) | Crude HR (95% CI) | Adjusted HR (95% CI) | *N* of event | Incidence rate (100 person- years) | Adjusted HR (95% CI) |
| **Bleeding** | | | | | | | |
| dipyridamole nonuser | 2,528 | 1.36 | 1.0 (reference) | 1.0 (reference) | 1,884 | 1.38 | 1.0 (reference) |
| dipyridamole user | 1,087 | 1.38 | 1.03 (0.96–1.11) | 0.99 (0.92–1.06) | 1,047 | 1.37 | 1.00 (0.92–1.08) |

Abbreviations: CI, confidence interval; CKD, chronic kidney disease; HR, hazard ratio
A multivariate analysis was adjusted for all variables listed in Table 1.
*$P < 0.05$ compared with dipyridamole nonusers.
